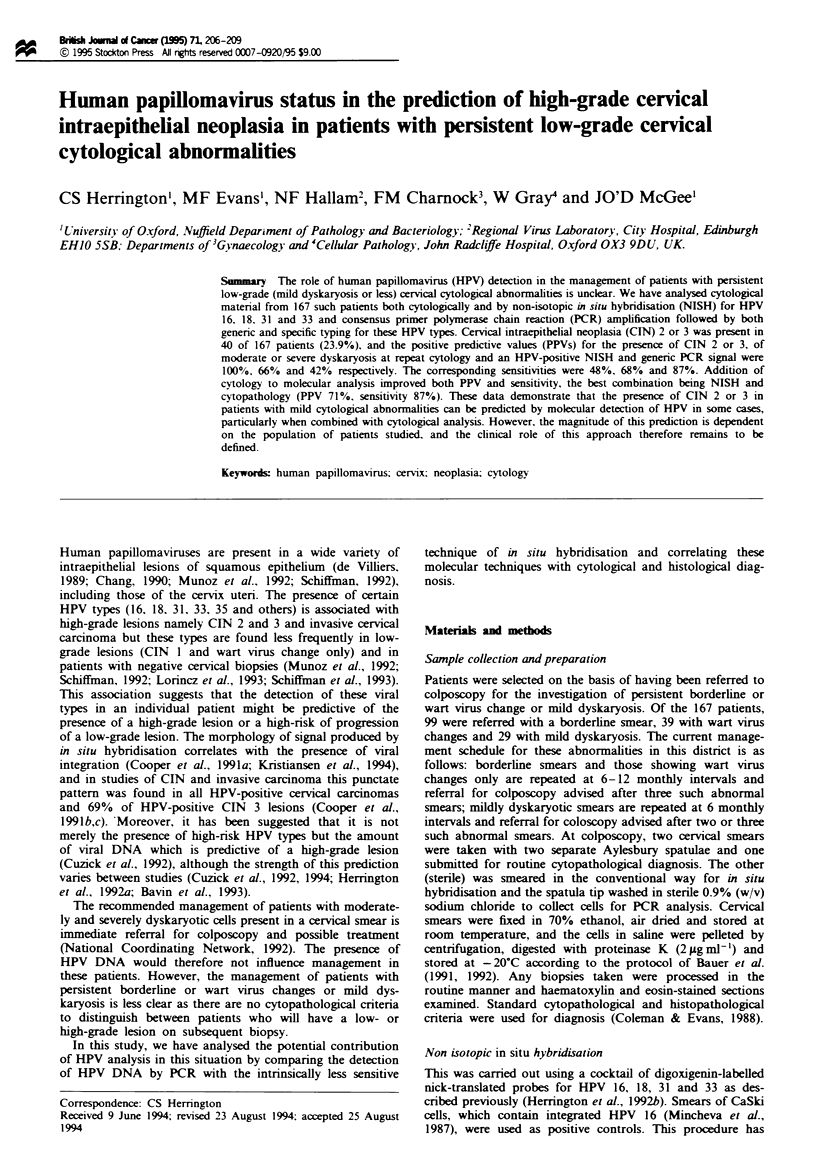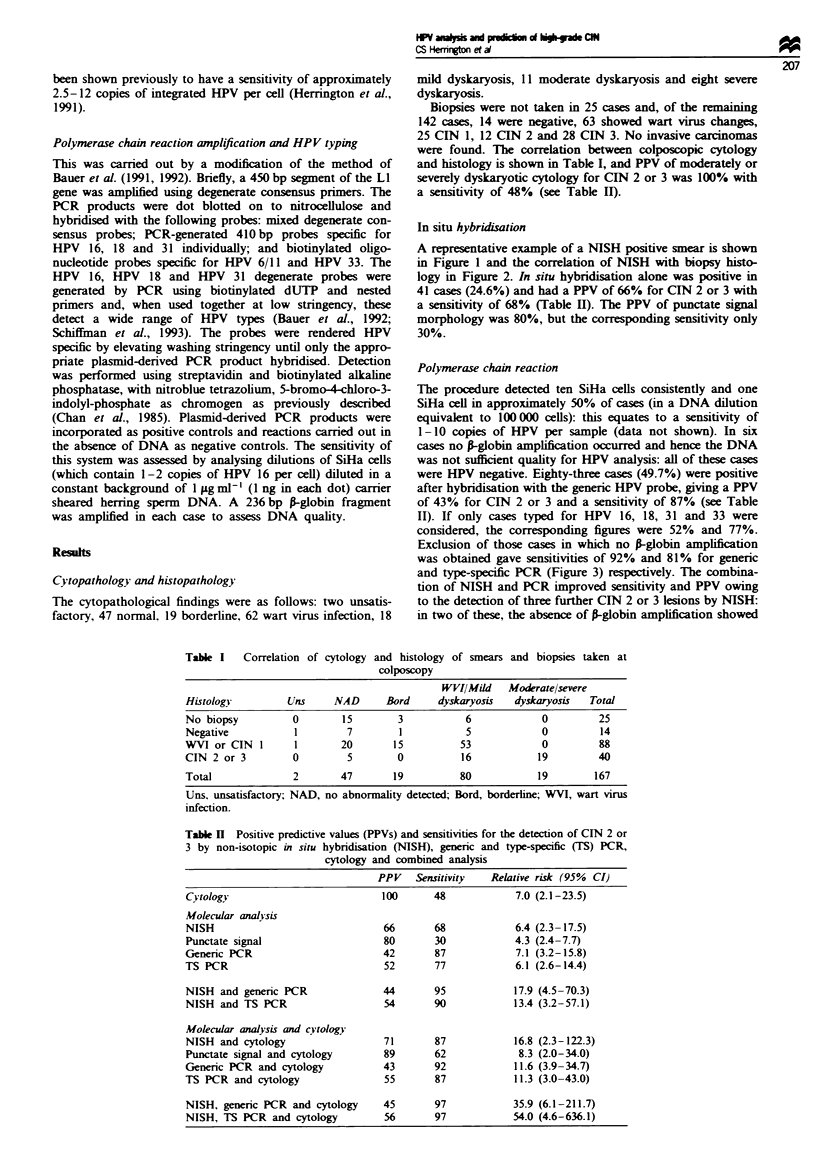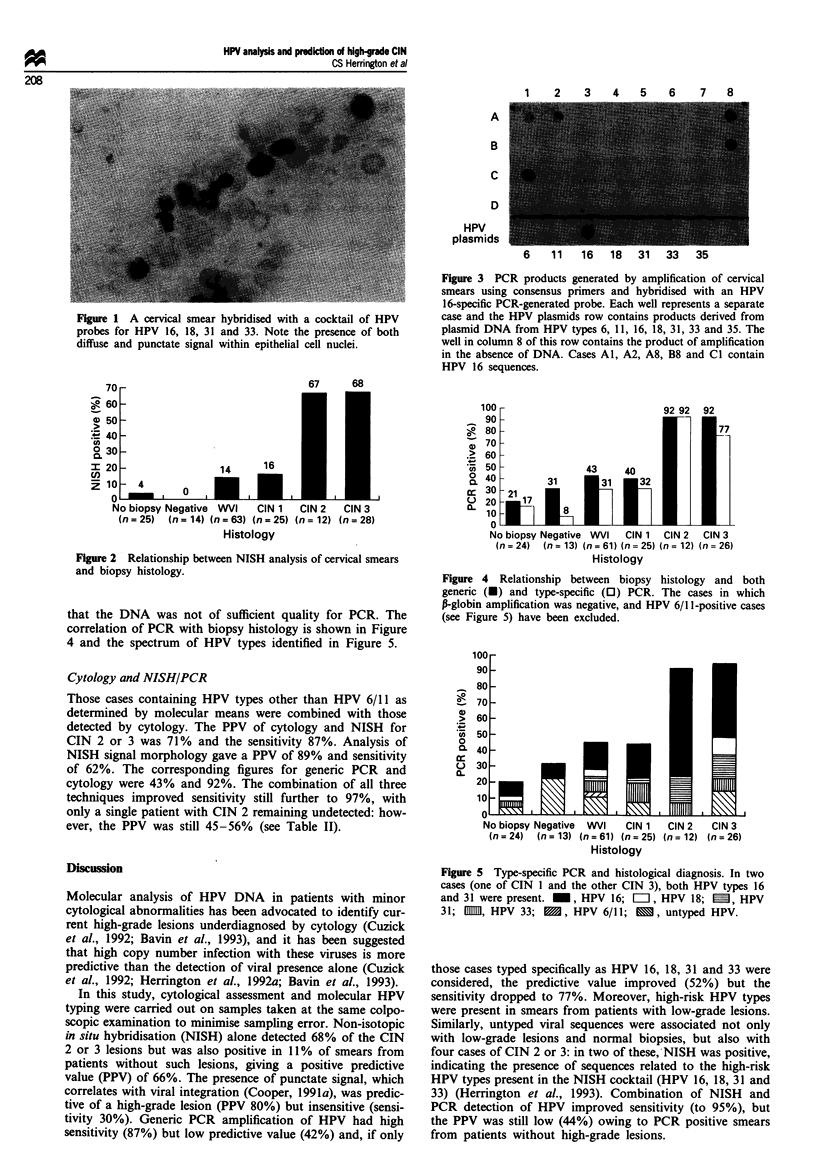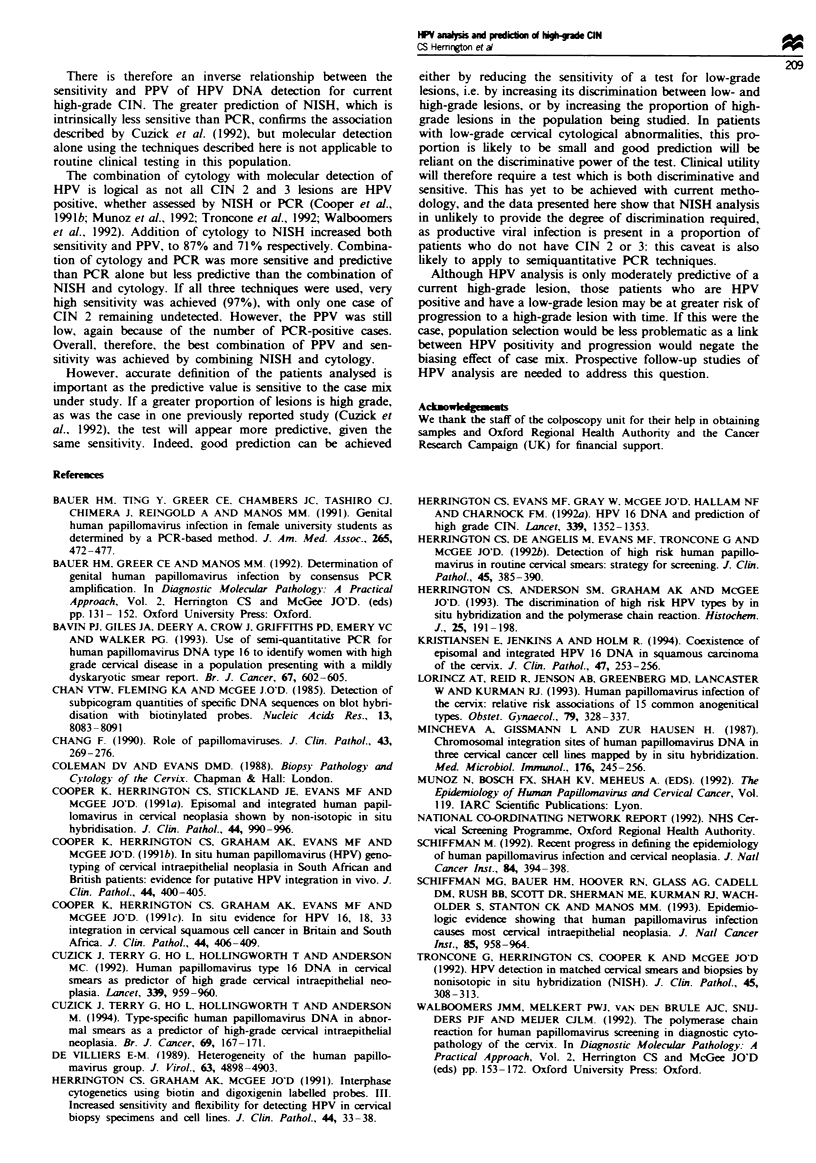# Human papillomavirus status in the prediction of high-grade cervical intraepithelial neoplasia in patients with persistent low-grade cervical cytological abnormalities.

**DOI:** 10.1038/bjc.1995.42

**Published:** 1995-01

**Authors:** C. S. Herrington, M. F. Evans, N. F. Hallam, F. M. Charnock, W. Gray, J. D. McGee

**Affiliations:** University of Oxford, Nuffield Department of Pathology and Bacteriology, UK.

## Abstract

**Images:**


British Journal of Cancer (1995) 71, 206-209

© 1995 Stockton Press All rights reserved 0007-0920/95 $9.00

### Human papillomavirus status in the prediction of high-grade cervical intraepithelial neoplasia in patients with persistent low-grade cervical cytological abnormalities

CS Herrington[1], MF Evans[1], NF Hallam[2], FM Charnock[3], W Gray[4] and JO'D McGee[1]

[1]*University of Oxford, Nuffield Department of Pathology and Bacteriology;* [2]*Regional Virus Laboratory, City Hospital, Edinburgh EH10 5SB; Departments of* [3]*Gynaecology and* [4]*Cellular Pathology, John Radcliffe Hospital, Oxford OX3 9DU, UK.*

**Summary** The role of human papillomavirus (HPV) detection in the management of patients with persistent low-grade (mild dyskaryosis or less) cervical cytological abnormalities is unclear. We have analysed cytological material from 167 such patients both cytologically and by non-isotopic *in situ* hybridisation (NISH) for HPV 16, 18, 31 and 33 and consensus primer polymerase chain reaction (PCR) amplification followed by both generic and specific typing for these HPV types. Cervical intraepithelial neoplasia (CIN) 2 or 3 was present in 40 of 167 patients (23.9%), and the positive predictive values (PPVs) for the presence of CIN 2 or 3, of moderate or severe dyskaryosis at repeat cytology and an HPV-positive NISH and generic PCR signal were 100%, 66% and 42% respectively. The corresponding sensitivities were 48%, 68% and 87%. Addition of cytology to molecular analysis improved both PPV and sensitivity, the best combination being NISH and cytopathology (PPV 71%, sensitivity 87%). These data demonstrate that the presence of CIN 2 or 3 in patients with mild cytological abnormalities can be predicted by molecular detection of HPV in some cases, particularly when combined with cytological analysis. However, the magnitude of this prediction is dependent on the population of patients studied, and the clinical role of this approach therefore remains to be defined.

**Keywords:** human papillomavirus; cervix; neoplasia; cytology

Human papillomaviruses are present in a wide variety of intraepithelial lesions of squamous epithelium (de Villiers, 1989; Chang, 1990; Munoz *et al.*, 1992; Schiffman, 1992), including those of the cervix uteri. The presence of certain HPV types (16, 18, 31, 33, 35 and others) is associated with high-grade lesions namely CIN 2 and 3 and invasive cervical carcinoma but these types are found less frequently in low-grade lesions (CIN 1 and wart virus change only) and in patients with negative cervical biopsies (Munoz *et al.*, 1992; Schiffman, 1992; Lorincz *et al.*, 1993; Schiffman *et al.*, 1993). This association suggests that the detection of these viral types in an individual patient might be predictive of the presence of a high-grade lesion or a high-risk of progression of a low-grade lesion. The morphology of signal produced by *in situ* hybridisation correlates with the presence of viral integration (Cooper *et al.*, 1991*a*; Kristiansen *et al.*, 1994), and in studies of CIN and invasive carcinoma this punctate pattern was found in all HPV-positive cervical carcinomas and 69% of HPV-positive CIN 3 lesions (Cooper *et al.*, 1991*b*,*c*). Moreover, it has been suggested that it is not merely the presence of high-risk HPV types but the amount of viral DNA which is predictive of a high-grade lesion (Cuzick *et al.*, 1992), although the strength of this prediction varies between studies (Cuzick *et al.*, 1992, 1994; Herrington *et al.*, 1992*a*; Bavin *et al.*, 1993).

The recommended management of patients with moderately and severely dyskaryotic cells present in a cervical smear is immediate referral for colposcopy and possible treatment (National Coordinating Network, 1992). The presence of HPV DNA would therefore not influence management in these patients. However, the management of patients with persistent borderline or wart virus changes or mild dyskaryosis is less clear as there are no cytopathological criteria to distinguish between patients who will have a low- or high-grade lesion on subsequent biopsy.

In this study, we have analysed the potential contribution of HPV analysis in this situation by comparing the detection of HPV DNA by PCR with the intrinsically less sensitive technique of *in situ* hybridisation and correlating these molecular techniques with cytological and histological diagnosis.

Correspondence: CS Herrington
Received 9 June 1994; revised 23 August 1994; accepted 25 August 1994

### Materials and methods

### *Sample collection and preparation*

Patients were selected on the basis of having been referred to colposcopy for the investigation of persistent borderline or wart virus change or mild dyskaryosis. Of the 167 patients, 99 were referred with a borderline smear, 39 with wart virus changes and 29 with mild dyskaryosis. The current management schedule for these abnormalities in this district is as follows: borderline smears and those showing wart virus changes only are repeated at 6-12 monthly intervals and referral for colposcopy advised after three such abnormal smears; mildly dyskaryotic smears are repeated at 6 monthly intervals and referral for coloscopy advised after two or three such abnormal smears. At colposcopy, two cervical smears were taken with two separate Aylesbury spatulae and one submitted for routine cytopathological diagnosis. The other (sterile) was smeared in the conventional way for *in situ* hybridisation and the spatula tip washed in sterile 0.9% (w/v) sodium chloride to collect cells for PCR analysis. Cervical smears were fixed in 70% ethanol, air dried and stored at room temperature, and the cells in saline were pelleted by centrifugation, digested with proteinase K ($2\,\mu g\,ml^{-1}$) and stored at $-20°C$ according to the protocol of Bauer *et al.* (1991, 1992). Any biopsies taken were processed in the routine manner and haematoxylin and eosin-stained sections examined. Standard cytopathological and histopathological criteria were used for diagnosis (Coleman & Evans, 1988).

### *Non isotopic* in situ *hybridisation*

This was carried out using a cocktail of digoxigenin-labelled nick-translated probes for HPV 16, 18, 31 and 33 as described previously (Herrington *et al.*, 1992*b*). Smears of CaSki cells, which contain integrated HPV 16 (Mincheva *et al.*, 1987), were used as positive controls. This procedure has been shown previously to have a sensitivity of approximately 2.5-12 copies of integrated HPV per cell (Herrington *et al.*, 1991).

*Polymerase chain reaction amplification and HPV typing*

This was carried out by a modification of the method of Bauer *et al.* (1991, 1992). Briefly, a 450 bp segment of the L1 gene was amplified using degenerate consensus primers. The PCR products were dot blotted on to nitrocellulose and hybridised with the following probes: mixed degenerate consensus probes; PCR-generated 410 bp probes specific for HPV 16, 18 and 31 individually; and biotinylated oligonucleotide probes specific for HPV 6/11 and HPV 33. The HPV 16, HPV 18 and HPV 31 degenerate probes were generated by PCR using biotinylated dUTP and nested primers and, when used together at low stringency, these detect a wide range of HPV types (Bauer *et al.*, 1992; Schiffman *et al.*, 1993). The probes were rendered HPV specific by elevating washing stringency until only the appropriate plasmid-derived PCR product hybridised. Detection was performed using streptavidin and biotinylated alkaline phosphatase, with nitroblue tetrazolium, 5-bromo-4-chloro-3-indolyl-phosphate as chromogen as previously described (Chan *et al.*, 1985). Plasmid-derived PCR products were incorporated as positive controls and reactions carried out in the absence of DNA as negative controls. The sensitivity of this system was assessed by analysing dilutions of SiHa cells (which contain 1-2 copies of HPV 16 per cell) diluted in a constant background of 1 $\mu g\ ml^{-1}$ (1 ng in each dot) carrier sheared herring sperm DNA. A 236 bp β-globin fragment was amplified in each case to assess DNA quality.

### Results

*Cytopathology and histopathology*

The cytopathological findings were as follows: two unsatisfactory, 47 normal, 19 borderline, 62 wart virus infection, 18 mild dyskaryosis, 11 moderate dyskaryosis and eight severe dyskaryosis.

Biopsies were not taken in 25 cases and, of the remaining 142 cases, 14 were negative, 63 showed wart virus changes, 25 CIN 1, 12 CIN 2 and 28 CIN 3. No invasive carcinomas were found. The correlation between colposcopic cytology and histology is shown in Table I, and PPV of moderately or severely dyskaryotic cytology for CIN 2 or 3 was 100% with a sensitivity of 48% (see Table II).

In situ *hybridisation*

A representative example of a NISH positive smear is shown in Figure 1 and the correlation of NISH with biopsy histology in Figure 2. *In situ* hybridisation alone was positive in 41 cases (24.6%) and had a PPV of 66% for CIN 2 or 3 with a sensitivity of 68% (Table II). The PPV of punctate signal morphology was 80%, but the corresponding sensitivity only 30%.

*Polymerase chain reaction*

The procedure detected ten SiHa cells consistently and one SiHa cell in approximately 50% of cases (in a DNA dilution equivalent to 100 000 cells): this equates to a sensitivity of 1-10 copies of HPV per sample (data not shown). In six cases no β-globin amplification occurred and hence the DNA was not sufficient quality for HPV analysis: all of these cases were HPV negative. Eighty-three cases (49.7%) were positive after hybridisation with the generic HPV probe, giving a PPV of 43% for CIN 2 or 3 and a sensitivity of 87% (see Table II). If only cases typed for HPV 16, 18, 31 and 33 were considered, the corresponding figures were 52% and 77%. Exclusion of those cases in which no β-globin amplification was obtained gave sensitivities of 92% and 81% for generic and type-specific PCR (Figure 3) respectively. The combination of NISH and PCR improved sensitivity and PPV owing to the detection of three further CIN 2 or 3 lesions by NISH: in two of these, the absence of β-globin amplification showed

**Table I** Correlation of cytology and histology of smears and biopsies taken at colposcopy

| *Histology* | *Uns* | *NAD* | *Bord* | *WVI/Mild dyskaryosis* | *Moderate/severe dyskaryosis* | *Total* |
|---|---|---|---|---|---|---|
| No biopsy | 0 | 15 | 3 | 6 | 0 | 25 |
| Negative | 1 | 7 | 1 | 5 | 0 | 14 |
| WVI or CIN 1 | 1 | 20 | 15 | 53 | 0 | 88 |
| CIN 2 or 3 | 0 | 5 | 0 | 16 | 19 | 40 |
| Total | 2 | 47 | 19 | 80 | 19 | 167 |

Uns, unsatisfactory; NAD, no abnormality detected; Bord, borderline; WVI, wart virus infection.

**Table II** Positive predictive values (PPVs) and sensitivities for the detection of CIN 2 or 3 by non-isotopic *in situ* hybridisation (NISH), generic and type-specific (TS) PCR, cytology and combined analysis

| | *PPV* | *Sensitivity* | *Relative risk (95% CI)* |
|---|---|---|---|
| *Cytology* | 100 | 48 | 7.0 (2.1-23.5) |
| *Molecular analysis* | | | |
| NISH | 66 | 68 | 6.4 (2.3-17.5) |
| Punctate signal | 80 | 30 | 4.3 (2.4-7.7) |
| Generic PCR | 42 | 87 | 7.1 (3.2-15.8) |
| TS PCR | 52 | 77 | 6.1 (2.6-14.4) |
| NISH and generic PCR | 44 | 95 | 17.9 (4.5-70.3) |
| NISH and TS PCR | 54 | 90 | 13.4 (3.2-57.1) |
| *Molecular analysis and cytology* | | | |
| NISH and cytology | 71 | 87 | 16.8 (2.3-122.3) |
| Punctate signal and cytology | 89 | 62 | 8.3 (2.0-34.0) |
| Generic PCR and cytology | 43 | 92 | 11.6 (3.9-34.7) |
| TS PCR and cytology | 55 | 87 | 11.3 (3.0-43.0) |
| NISH, generic PCR and cytology | 45 | 97 | 35.9 (6.1-211.7) |
| NISH, TS PCR and cytology | 56 | 97 | 54.0 (4.6-636.1) |

**Figure 1** A cervical smear hybridised with a cocktail of HPV probes for HPV 16, 18, 31 and 33. Note the presence of both diffuse and punctate signal within epithelial cell nuclei.

**Figure 2** Relationship between NISH analysis of cervical smears and biopsy histology.

that the DNA was not of sufficient quality for PCR. The correlation of PCR with biopsy histology is shown in Figure 4 and the spectrum of HPV types identified in Figure 5.

*Cytology and NISH/PCR*

Those cases containing HPV types other than HPV 6/11 as determined by molecular means were combined with those detected by cytology. The PPV of cytology and NISH for CIN 2 or 3 was 71% and the sensitivity 87%. Analysis of NISH signal morphology gave a PPV of 89% and sensitivity of 62%. The corresponding figures for generic PCR and cytology were 43% and 92%. The combination of all three techniques improved sensitivity still further to 97%, with only a single patient with CIN 2 remaining undetected: however, the PPV was still 45–56% (see Table II).

### Discussion

Molecular analysis of HPV DNA in patients with minor cytological abnormalities has been advocated to identify current high-grade lesions underdiagnosed by cytology (Cuzick *et al.*, 1992; Bavin *et al.*, 1993), and it has been suggested that high copy number infection with these viruses is more predictive than the detection of viral presence alone (Cuzick *et al.*, 1992; Herrington *et al.*, 1992*a*; Bavin *et al.*, 1993).

In this study, cytological assessment and molecular HPV typing were carried out on samples taken at the same colposcopic examination to minimise sampling error. Non-isotopic *in situ* hybridisation (NISH) alone detected 68% of the CIN 2 or 3 lesions but was also positive in 11% of smears from patients without such lesions, giving a positive predictive value (PPV) of 66%. The presence of punctate signal, which correlates with viral integration (Cooper, 1991*a*), was predictive of a high-grade lesion (PPV 80%) but insensitive (sensitivity 30%). Generic PCR amplification of HPV had high sensitivity (87%) but low predictive value (42%) and, if only those cases typed specifically as HPV 16, 18, 31 and 33 were considered, the predictive value improved (52%) but the sensitivity dropped to 77%. Moreover, high-risk HPV types were present in smears from patients with low-grade lesions. Similarly, untyped viral sequences were associated not only with low-grade lesions and normal biopsies, but also with four cases of CIN 2 or 3: in two of these, NISH was positive, indicating the presence of sequences related to the high-risk HPV types present in the NISH cocktail (HPV 16, 18, 31 and 33) (Herrington *et al.*, 1993). Combination of NISH and PCR detection of HPV improved sensitivity (to 95%), but the PPV was still low (44%) owing to PCR positive smears from patients without high-grade lesions.

**Figure 3** PCR products generated by amplification of cervical smears using consensus primers and hybridised with an HPV 16-specific PCR-generated probe. Each well represents a separate case and the HPV plasmids row contains products derived from plasmid DNA from HPV types 6, 11, 16, 18, 31, 33 and 35. The well in column 8 of this row contains the product of amplification in the absence of DNA. Cases A1, A2, A8, B8 and C1 contain HPV 16 sequences.

**Figure 4** Relationship between biopsy histology and both generic (■) and type-specific (□) PCR. The cases in which β-globin amplification was negative, and HPV 6/11-positive cases (see Figure 5) have been excluded.

**Figure 5** Type-specific PCR and histological diagnosis. In two cases (one of CIN 1 and the other CIN 3), both HPV types 16 and 31 were present. ■, HPV 16; □, HPV 18; ▤, HPV 31; ▥, HPV 33; ▨, HPV 6/11; ▧, untyped HPV.

There is therefore an inverse relationship between the sensitivity and PPV of HPV DNA detection for current high-grade CIN. The greater prediction of NISH, which is intrinsically less sensitive than PCR, confirms the association described by Cuzick *et al.* (1992), but molecular detection alone using the techniques described here is not applicable to routine clinical testing in this population.

The combination of cytology with molecular detection of HPV is logical as not all CIN 2 and 3 lesions are HPV positive, whether assessed by NISH or PCR (Cooper *et al.*, 1991*b*; Munoz *et al.*, 1992; Troncone *et al.*, 1992; Walboomers *et al.*, 1992). Addition of cytology to NISH increased both sensitivity and PPV, to 87% and 71% respectively. Combination of cytology and PCR was more sensitive and predictive than PCR alone but less predictive than the combination of NISH and cytology. If all three techniques were used, very high sensitivity was achieved (97%), with only one case of CIN 2 remaining undetected. However, the PPV was still low, again because of the number of PCR-positive cases. Overall, therefore, the best combination of PPV and sensitivity was achieved by combining NISH and cytology.

However, accurate definition of the patients analysed is important as the predictive value is sensitive to the case mix under study. If a greater proportion of lesions is high grade, as was the case in one previously reported study (Cuzick *et al.*, 1992), the test will appear more predictive, given the same sensitivity. Indeed, good prediction can be achieved either by reducing the sensitivity of a test for low-grade lesions, i.e. by increasing its discrimination between low- and high-grade lesions, or by increasing the proportion of high-grade lesions in the population being studied. In patients with low-grade cervical cytological abnormalities, this proportion is likely to be small and good prediction will be reliant on the discriminative power of the test. Clinical utility will therefore require a test which is both discriminative and sensitive. This has yet to be achieved with current methodology, and the data presented here show that NISH analysis in unlikely to provide the degree of discrimination required, as productive viral infection is present in a proportion of patients who do not have CIN 2 or 3: this caveat is also likely to apply to semiquantitative PCR techniques.

Although HPV analysis is only moderately predictive of a current high-grade lesion, those patients who are HPV positive and have a low-grade lesion may be at greater risk of progression to a high-grade lesion with time. If this were the case, population selection would be less problematic as a link between HPV positivity and progression would negate the biasing effect of case mix. Prospective follow-up studies of HPV analysis are needed to address this question.

**Acknowledgements**
We thank the staff of the colposcopy unit for their help in obtaining samples and Oxford Regional Health Authority and the Cancer Research Campaign (UK) for financial support.

**References**

BAUER HM, TING Y, GREER CE, CHAMBERS JC, TASHIRO CJ, CHIMERA J, REINGOLD A AND MANOS MM. (1991). Genital human papillomavirus infection in female university students as determined by a PCR-based method. *J. Am. Med. Assoc.*, **265**, 472–477.

BAUER HM, GREER CE AND MANOS MM. (1992). Determination of genital human papillomavirus infection by consensus PCR amplification. In *Diagnostic Molecular Pathology: A Practical Approach*, Vol. 2, Herrington CS and McGee JO'D. (eds) pp. 131–152. Oxford University Press: Oxford.

BAVIN PJ, GILES JA, DEERY A, CROW J, GRIFFITHS PD, EMERY VC AND WALKER PG. (1993). Use of semi-quantitative PCR for human papillomavirus DNA type 16 to identify women with high grade cervical disease in a population presenting with a mildly dyskaryotic smear report. *Br. J. Cancer*, **67**, 602–605.

CHAN VTW, FLEMING KA AND McGEE J.O'D. (1985). Detection of subpicogram quantities of specific DNA sequences on blot hybridisation with biotinylated probes. *Nucleic Acids Res.*, **13**, 8083–8091

CHANG F. (1990). Role of papillomaviruses. *J. Clin. Pathol.*, **43**, 269–276.

COLEMAN DV AND EVANS DMD. (1988). *Biopsy Pathology and Cytology of the Cervix*. Chapman & Hall: London.

COOPER K, HERRINGTON CS, STICKLAND JE, EVANS MF AND McGEE JO'D. (1991*a*). Episomal and integrated human papillomavirus in cervical neoplasia shown by non-isotopic in situ hybridisation. *J. Clin. Pathol.*, **44**, 990–996.

COOPER K, HERRINGTON CS, GRAHAM AK, EVANS MF AND McGEE JO'D. (1991*b*). In situ human papillomavirus (HPV) genotyping of cervical intraepithelial neoplasia in South African and British patients: evidence for putative HPV integration in vivo. *J. Clin. Pathol.*, **44**, 400–405.

COOPER K, HERRINGTON CS, GRAHAM AK, EVANS MF AND McGEE JO'D. (1991*c*). In situ evidence for HPV 16, 18, 33 integration in cervical squamous cell cancer in Britain and South Africa. *J. Clin. Pathol.*, **44**, 406–409.

CUZICK J, TERRY G, HO L, HOLLINGWORTH T AND ANDERSON MC. (1992). Human papillomavirus type 16 DNA in cervical smears as predictor of high grade cervical intraepithelial neoplasia. *Lancet*, **339**, 959–960.

CUZICK J, TERRY G, HO L, HOLLINGWORTH T AND ANDERSON M. (1994). Type-specific human papillomavirus DNA in abnormal smears as a predictor of high-grade cervical intraepithelial neoplasia. *Br. J. Cancer*, **69**, 167–171.

DE VILLIERS E-M. (1989). Heterogeneity of the human papillomavirus group. *J. Virol.*, **63**, 4898–4903.

HERRINGTON CS, GRAHAM AK, McGEE JO'D (1991). Interphase cytogenetics using biotin and digoxigenin labelled probes. III. Increased sensitivity and flexibility for detecting HPV in cervical biopsy specimens and cell lines. *J. Clin. Pathol.*, **44**, 33–38.

HERRINGTON CS, EVANS MF, GRAY W, McGEE JO'D, HALLAM NF AND CHARNOCK FM. (1992*a*). HPV 16 DNA and prediction of high grade CIN. *Lancet*, **339**, 1352–1353.

HERRINGTON CS, DE ANGELIS M, EVANS MF, TRONCONE G AND McGEE JO'D. (1992*b*). Detection of high risk human papillomavirus in routine cervical smears: strategy for screening. *J. Clin. Pathol.*, **45**, 385–390.

HERRINGTON CS, ANDERSON SM, GRAHAM AK AND McGEE JO'D. (1993). The discrimination of high risk HPV types by in situ hybridization and the polymerase chain reaction. *Histochem. J.*, **25**, 191–198.

KRISTIANSEN E, JENKINS A AND HOLM R. (1994). Coexistence of episomal and integrated HPV 16 DNA in squamous carcinoma of the cervix. *J. Clin. Pathol.*, **47**, 253–256.

LORINCZ AT, REID R, JENSON AB, GREENBERG MD, LANCASTER W AND KURMAN RJ. (1993). Human papillomavirus infection of the cervix: relative risk associations of 15 common anogenitical types. *Obstet. Gynaecol.*, **79**, 328–337.

MINCHEVA A, GISSMANN L AND ZUR HAUSEN H. (1987). Chromosomal integration sites of human papillomavirus DNA in three cervical cancer cell lines mapped by in situ hybridization. *Med. Microbiol. Immunol.*, **176**, 245–256.

MUNOZ N, BOSCH FX, SHAH KV, MEHEUS A. (EDS). (1992). *The Epidemiology of Human Papillomavirus and Cervical Cancer*, Vol. 119. IARC Scientific Publications: Lyon.

NATIONAL CO-ORDINATING NETWORK REPORT (1992). NHS Cervical Screening Programme, Oxford Regional Health Authority.

SCHIFFMAN M. (1992). Recent progress in defining the epidemiology of human papillomavirus infection and cervical neoplasia. *J. Natl Cancer Inst.*, **84**, 394–398.

SCHIFFMAN MG, BAUER HM, HOOVER RN, GLASS AG, CADELL DM, RUSH BB, SCOTT DR, SHERMAN ME, KURMAN RJ, WACHOLDER S, STANTON CK AND MANOS MM. (1993). Epidemiologic evidence showing that human papillomavirus infection causes most cervical intraepithelial neoplasia. *J. Natl Cancer Inst.*, **85**, 958–964.

TRONCONE G, HERRINGTON CS, COOPER K AND McGEE JO'D (1992). HPV detection in matched cervical smears and biopsies by nonisotopic in situ hybridization (NISH). *J. Clin. Pathol.*, **45**, 308–313.

WALBOOMERS JMM, MELKERT PWJ, VAN DEN BRULE AJC, SNIJDERS PJF AND MEIJER CJLM. (1992). The polymerase chain reaction for human papillomavirus screening in diagnostic cytopathology of the cervix. In *Diagnostic Molecular Pathology: A Practical Approach*, Vol. 2, Herrington CS and McGee JO'D (eds) pp. 153–172. Oxford University Press: Oxford.